# P-1187. Comparative Evaluation of Clinical Characteristics in Pediatric Patients Hospitalized in Taiwan with RSV and hMPV Infections

**DOI:** 10.1093/ofid/ofae631.1371

**Published:** 2025-01-29

**Authors:** Yu-Ting chiu, Hsiu-Mei Wei, Yu-Lung Hsu, Yu-Chang Chang, Chiung-Tzu Hsiao, Huan-Cheng Lai, Jiun-An Chen, Yan-Yi Low, Hsiao-Chuan Lin, Kao-Pin Hwang

**Affiliations:** China Medical University Children’s Hospital, China Medical University, Taichung, Taichung, Taiwan (Republic of China); China Medical University Children’s Hospital, China Medical University, Taichung, Taichung, Taiwan (Republic of China); China Medical University Children’s Hospital, China Medical University, Taichung, Taichung, Taiwan (Republic of China); Department of Laboratory Medicine, China Medical University Hospital, Taichung, Taiwan, Taichung City, Taichung, Taiwan; China Medical University Hospital, Taichung, Taichung, Taiwan; China Medical University Children’s Hospital, China Medical University, Taichung, Taichung, Taiwan (Republic of China); China Medical University Children’s Hospital, China Medical University, Taichung, Taichung, Taiwan (Republic of China); China Medical University Children’s Hospital, China Medical University, Taichung, Taichung, Taiwan (Republic of China); China Medical University Children’s Hospital, China Medical University, Taichung, Taichung, Taiwan (Republic of China); China Medical University Children’s Hospital, China Medical University, Taichung, Taichung, Taiwan (Republic of China)

## Abstract

**Background:**

Respiratory Syncytial Virus (RSV) and Human Metapneumovirus (hMPV) are prevalent pathogens responsible for respiratory tract infections in infants and young children. This study aims to conduct a comprehensive analysis of the clinical presentations and outcomes among hospitalized infants and young children with RSV and hMPV infections.

**Figure:**
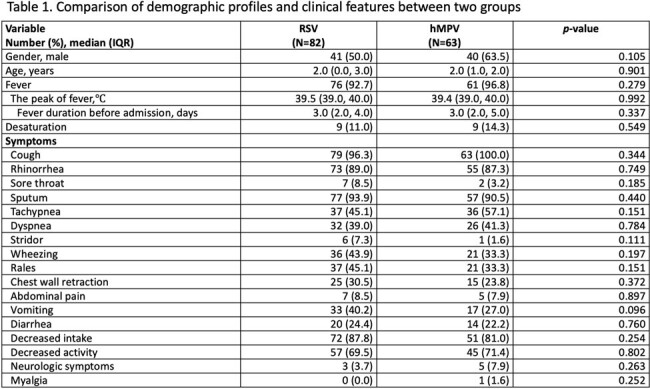

**Methods:**

This retrospective observational study was conducted at China Medical University Children's Hospital (CMUCH) from January 2020 to December 2021. The study enrolled patients under 5 years old admitted due to respiratory tract infections, with RSV or hMPV detection confirmed using a commercial respiratory panel employing real-time polymerase chain reaction. Demographic details, clinical manifestations, and outcomes were gathered and analyzed to facilitate comparative assessments.

**Figure:**
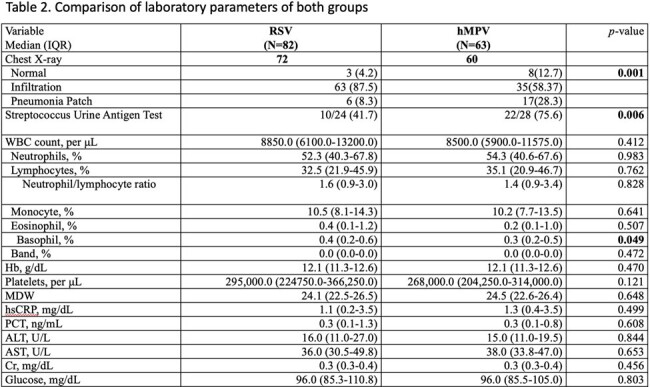

**Results:**

A total of 145 pediatric patients were included in the study. Among them, 82 patients tested positive for RSV, while 63 patients tested positive for hMPV. There were no significant differences in age and gender distribution between the two groups. Common symptoms such as fever, cough, and rhinorrhea were observed in both groups without notable disparities. No significant differences were found in laboratory parameters between the groups. Pneumonia patches were more frequently observed in the hMPV group and less frequently observed in the RSV group (28.3% vs. 8.3%, *p*=0.001). Medical interventions did not significantly differ between the two groups. However, the hMPV group exhibited a notably higher rate of antibiotic usage compared to the RSV group (90.5% vs. 57.3%, *p*<0.001). Moreover, there were no significant discrepancies in the duration of hospitalization (5 vs. 5 days, *p*=0.110), requirement for oxygen (36.6% vs. 25.4%, *p*=0.151), and admission to the intensive care unit (ICU) (14.6% vs. 9.5%, *p*=0.355) between the two groups. Only one RSV patient required intubation, and there were no reported fatalities in either group during the study period.

**Figure:**
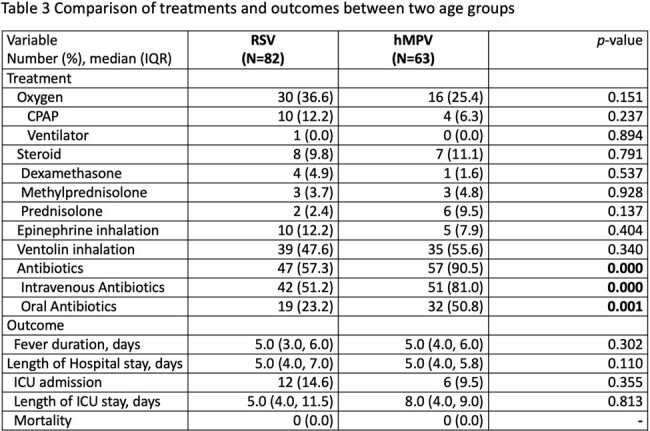

**Conclusion:**

The findings indicate that RSV and hMPV infections have similar clinical presentations, highlighting diagnostic challenges in the absence of molecular methods. Patients with hMPV are more likely to develop pneumonia patches and receive antibiotic therapy.

**Disclosures:**

**All Authors**: No reported disclosures

